# Isolation of immune-regulatory *Tetragenococcus halophilus* from miso

**DOI:** 10.1371/journal.pone.0208821

**Published:** 2018-12-26

**Authors:** Toshihiko Kumazawa, Atsuhisa Nishimura, Noriyuki Asai, Takahiro Adachi

**Affiliations:** 1 Ichibiki CO., LTD. Nagoya, Japan; 2 Department of Immunology, Medical Research Institute, Tokyo Medical and Dental University, Tokyo, Japan; The University of Tokyo, JAPAN

## Abstract

*Tetragenococcus halophilus* is a halophilic lactic acid bacterium that exists in the traditional Japanese seasoning miso—a fermented soy paste. Considering the popularity of miso as a component of healthy diet, we attempted to evaluate the immunoregulatory functions of *T*. *halophilus* spices isolated from miso. We screened 56 strains that facilitated the upregulation of activation markers such as CD86 and CD69 on B cells and T cells *in vitro*. Of these, 7 strains (Nos. 1, 3, 13, 15, 19, 30, and 31) were found to preferentially induce the CD86 expression on B cells. Furthermore, DNA microarray analysis revealed that *T*. *halophilus* strain No. 1 significantly augmented the gene expressions of CD86, CD70, IL-10, INF-γ, and IL-22 in B cells. We confirmed these results at the protein level by flow cytometry. Mice feeding diet containing 1% *T*. *halophilus* No. 1 exhibited significantly greater IgA production in the serum. Furthermore, a diet containing 1% *T*. *halophilus* No. 1 augmented ovoalbumin (OVA)-specific IgG titer in mice upon OVA/alum immunization. Thus, we demonstrated that *T*. *halophilus* No. 1 is a strong immunomodulatory strain with potential as a probiotic.

## Introduction

Probiotic bacteria impart beneficial effects on health [1–5]. Several strains of lactic acid bacteria (LAB) have been identified as probiotics. LAB are utilized in the preparation of several fermented foods such as lactic beverage, yogurt, cheese, and pickles. LAB are also major constituents of small intestinal commensal flora [6]. The oral administration of some LAB strains has been shown to exhibit diverse physical activities, including the stimulation of innate immunity at the mucosal sites and systemic immune responses against pathogenic bacteria or viruses [2, 7–12].

LAB are abundant in miso, soy sauce, and pickles of the Japanese traditional diet Washoku. Washoku—the Japanese traditional dietary culture—has been assigned to the Representative List of the Intangible Cultural Heritage of Humanity and is considered to be a healthy diet [13]. Washoku consists of rice and miso soup with some fish and vegetables. Miso soup is a typical Japanese traditional fermented food and, recently, its beneficial effect on the human health was documented [13–16].

Miso, which is a Japanese traditional fermented ingredient of soy paste, is of three main types: bean miso, rice miso, and barley miso, based on the materials of the molts. Bean miso is made from soybeans, salt, water, and *Aspergillus oryzae* together with some strains of LAB and yeast during the process of the brewage [17]. The LAB in miso belong to *T*. *halophilus*, which are salt-tolerant bacteria. The nutritive value of miso is excellent due to its abundant amino acids and vitamins. Furthermore, miso is a beneficial food for human health. Miso lowers the risk of cancer, hypertension, inflammation, lifestyle-related diseases, and prevents aging [14–16, 18–22]. Recently, *T*. *halophilus* derived from miso or soy sauce was shown to regulate immune cells such as dendritic cells and T cells. The administration of heat-killed LAB augments the levels of cytokines such as IL-12, IL-10, and IFN-γ and suppresses the levels of IL-4 and IL-5 [23–25]. Furthermore, *T*. *thermophilus* has been shown to augment the IL-10 and IFN-β production in dendritic cells [26].

Several LAB have been shown to possess immunoregulatory functions, and their biological activities for the host immune system remains unclear. The soluble form of immunoglobulins in the serum, mucosal barrier, saliva, tears, and milk account for one of the major adaptive immune responses. Although B cells differentiate into plasma cells, which produce immunoglobulins, the effect of probiotics on B cells is limited. In this study, we screened over 50 strains of miso-derived *T*. *halophilus* for B cell regulatory functions and identified a novel immunomodulatory function.

## Materials and methods

### Ethics statement

All mice were maintained in our animal facility under SPF conditions in accordance with guidelines of the Institutional Animal Care and Use Committee of Tokyo Medical and Dental University. All experimental procedures on animals were approved by the Institutional Animal Care and Use Committee of Tokyo Medical and Dental University (No. A2018-432), and all experiments were carried out in accordance with approved guidelines.

### Bacteria

A total of 56 strains of *T*. *halophilus* isolated from miso were cultured in 10SG10N medium (10% soy sauce, 10% NaCl, 1% glucose, 1% yeast extract, 0.5% polypeptone, 0.2% sodium acetate trihydrate, 0.02% MgSO_4_·7H_2_O, 0.001% MnSO_4_·4H_2_O, 0.001% FeSO_4_·7H_2_O, and 0.0025% Tween 80; pH 6.8) at 30°C for 4–7 days. Cultures were sterilized by autoclaving at 121°C for 15 min. Then, bacteria were collected by centrifugation, washed thrice with water, and then lyophilized. Freeze-dried bacterial cells were suspended in PBS.

### PCR amplification and sequencing of bacterial 16S rDNA

*T*. *halophilus* strains isolated from miso were cultured in 10SG10N medium, and total DNA was extracted using by NucleoSpin Microbial DNA (MACHEREY-NAGEL GmbH & Co. KG). The genome of bacterial 16S rDNA was amplified by PCR using the primers 10F (5’-GTT TGA TCC TGG CTC A-3’) and 1500R (5’-TAC CTT GTT ACG ACT T-3’). PCR products were purified by FastGene Gel/PCR Extraction Kit (NIPPON Genetics Co., Ltd). The purified PCR products were sequenced by Fasmac Co., Ltd., JAPAN, using Genetic Analyzer (Applied Biosystems 3130 XL, Switzerland). Database search and comparisons were done with the BLAST database.

### Cells and mice

The spleen cells of C57BL/6 mice were prepared as described previously [27]. B220^+^ B cells were isolated from the spleen cells using the BD IMag Cell Separation System according to the manufacturer’s instructions (Becton, Dickinson and Company).

C57BL/6 mice (8-week-old) were fed either a standard control diet, CE2 (Japan Crea), or a diet supplemented with 1% heat-killed *T*. *halophilus* for 2 weeks under specific pathogen free conditions.

Mice were immunized with 0.3 ml of OVA/alum (OVA: 50 μg) in PBS intraperitoneally. As a secondary immunization, mice were immunized with 0.3 ml of OVA/alum (OVA: 30 μg) in PBS intraperitoneally.

### *In vitro* immunological assay

A total of 1 × 10^6^ spleen cells were cultured in 1 mL of RPMI1640 medium containing 10% FCS with or without 1 μg of *T*. *halophilus* for 2 days. The activation cell surface markers CD69 and CD86 on spleen cells was evaluated by flow cytometry.

### Cytokine assay

The spleen cells were cultured for 2 days at a concentration of 1 × 10^6^ cells/mL in RPMI 1640 medium containing 10% FCS with or without 10 μμg of *T*. *halophilus*. BD GolgiStop (according to the manufacturer’s instructions; Becton, Dickinson and Company) was added to the medium at 6 h before the end of cultivation period. To measure the intracellular cytokines, BD Fixation/Permeabilization Solution Kit (Becton, Dickinson and Company) was used. Then, permeabilized cells were treated with APC-labeled anti-IL-10 antibodies (clone; JES5-16E3, BioLegend), Alexa Fluor 647-labeled anti-INF-γ antibodies (clone; XMG1.2; BD Pharmingen), and PE-labeled anti-IL-22 antibodies (clone; 1H8PWSR; eBioscience). Cells were analyzed by flow cytometry.

### Flow cytometry

The cells were analyzed on the MACSQuant Flow Cytometer (MiltenyiBiotec) using the following specific antibodies: VioletFluo 450-labeled anti-B220 antibodies (clone; RA3-6B2) and APC-labeled anti-CD86 antibodies (clone; GL-1) purchased from TONBO biosciences and Brilliant Violet 510 anti-mouse CD4 antibodies (clone; RM4-5) and phycoerythrin (PE)-labeled anti-CD69 antibodies (clone; H1.2F3) purchased from BioLegend. Dead cells were excluded by propidium iodide staining. Data analysis was conducted with FlowJo (FLOWJO, LLC). When detecting CD19 instead of B220, VioletFluo 450-labeled anti-CD19 antibodies (clone; 1D3, TONBO biosciences) were used.

### Measurement of the immunoglobulin levels

The immunoglobulin levels were measured as described previously [28] using enzyme-linked immunosorbent assays (ELISAs) by using the following antibodies: anti-IgM, anti-IgG, anti-IgA, and alkaline phosphatase-conjugated anti-IgM, anti-IgG, and anti-IgA (Southern Biotech). OVA-specific IgG was measured as described previously [28].

### DNA microarray

A total of 4 × 10^7^ spleen cells were cultured in 4 mL of RPMI1640 medium containing 10% FCS with or without 8 μg of *T*. *halophilus* for 2 days. B220^+^ B cells were isolated from the spleen cells using the BD IMag Cell Separation System (Becton, Dickinson and Company). Total RNAs were prepared from B cells using ISOGEN II (NIPPON GENE). The gene expression analysis was performed by DNA microarray. The measurement was entrusted to Macrogen JAPAN. DNA microarray analysis used the SurePrint G3 Mouse Gene Expression 8x60K (Agilent Technologies). Finally, the data were analyzed by the genetic manifested software R version 2.15.1.

### Statistical analysis

Experimental data are indicated as the mean ± standard deviations (S.D.). Statistical significance was evaluated by a two-tailed Student’s t test for unpaired data. P values < 0.05 were considered to be statistically significant.

## Results

### Screening of *T*. *halophilus* strains from miso in an *in vitro* immunological assay

**Fig 1:**
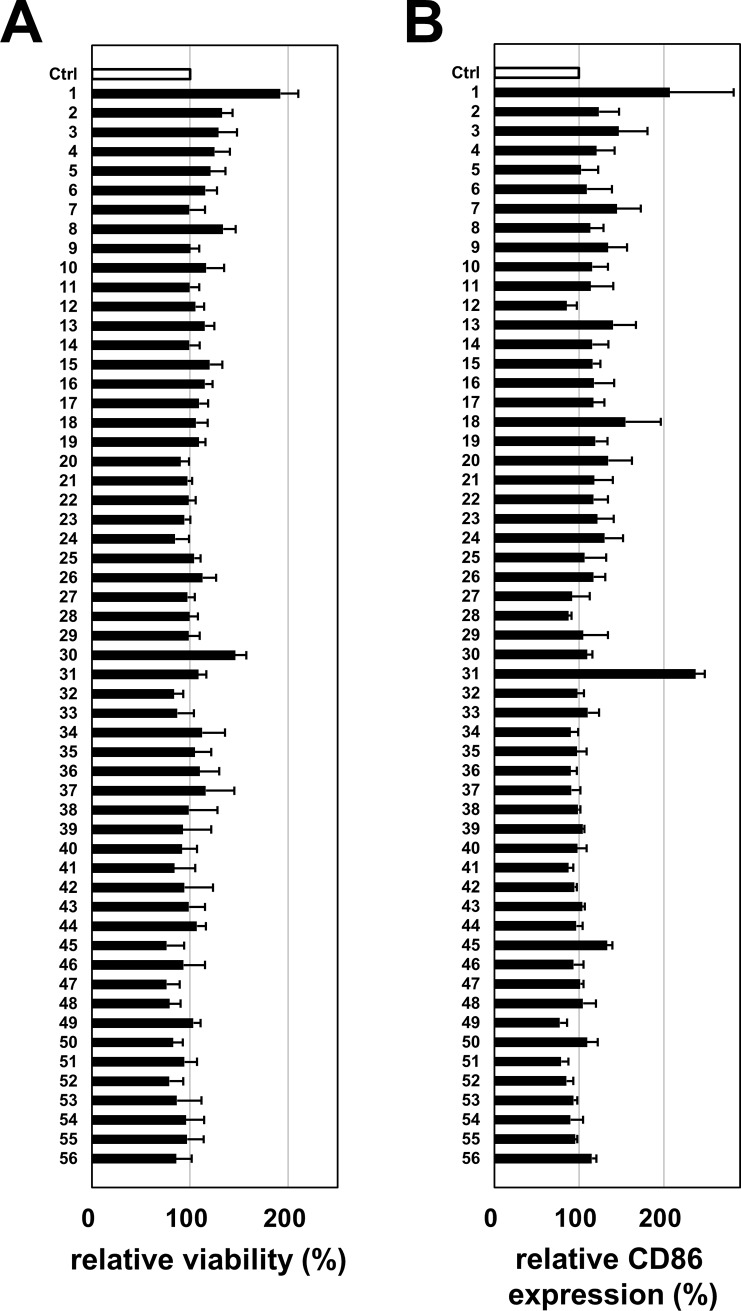
The CD86 expression on B cells cultured with *T*. *halophilus*. The spleen B220^+^ cells prepared from C57BL/6 mouse spleen were cultured with 1 μg of *T*. *halophilus* strains in 1 mL of PRMI1640 medium containing 10% FCS for 2 days. The cells were collected and stained with anti-B220 mAb and anti-CD86 mAb. Dead cells were stained with PI. The cells were analyzed by flow cytometry. Viability (A) and CD86^+^ cells of B220^+^cells (B) cultured without *T*. *halophilus* as a control was defined as 100%. Based on this parameter, the relative viability of cells and the relative CD86 expression cultured with *T*. *halophilus* were calculated. Bars indicate mean ± S.D. (n = 6).

**Fig 2:**
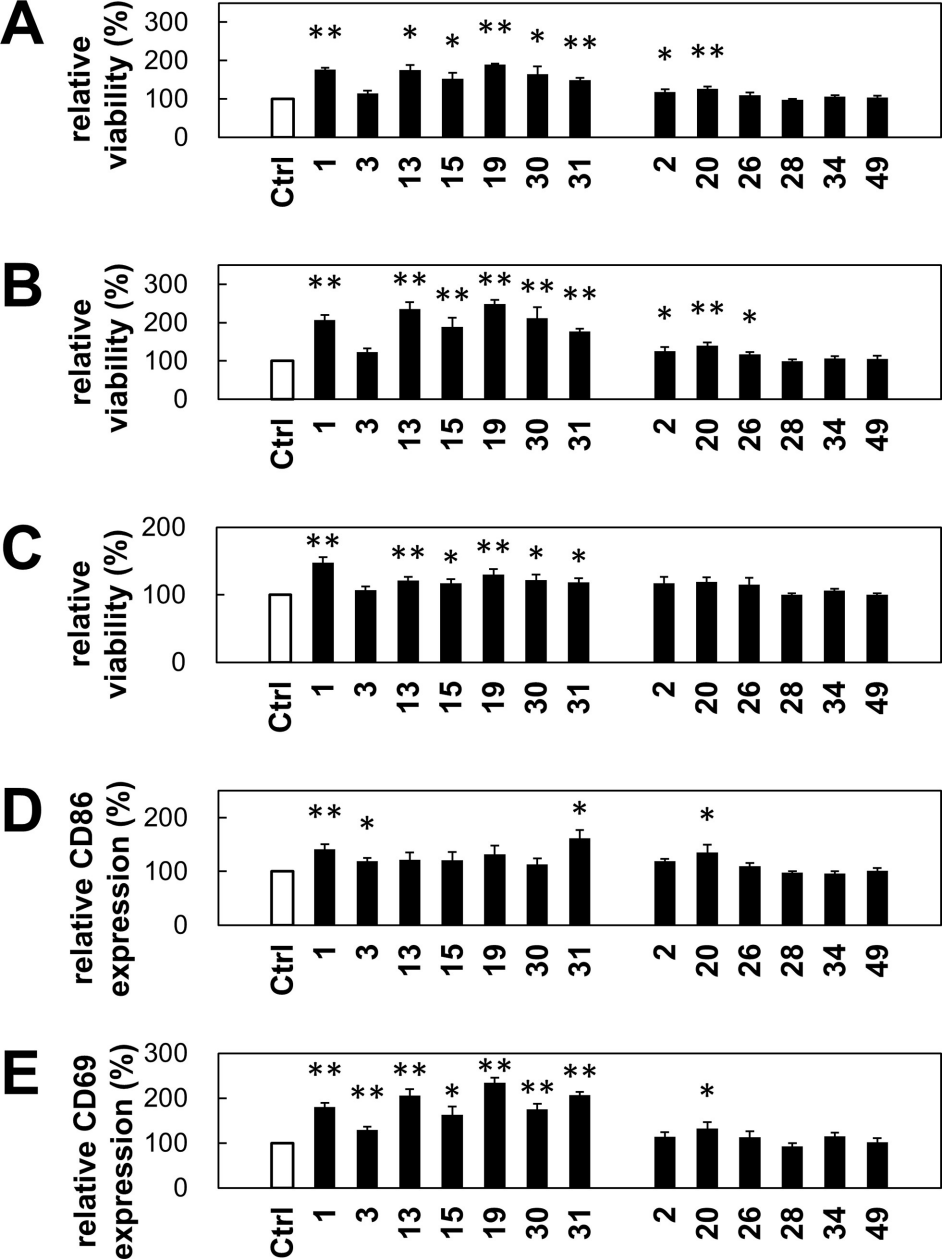
*T*. *halophilus-*mediated CD86 and CD69 expression on B cells and T cells, respectively. The spleen cells from C57BL/6 mice were cultured with 1 μg of *T*. *halophilus* strains in 1 mL of PRMI1640 medium containing 10% FCS for 2 days. The cells were collected and stained with anti-B220 mAb, anti-CD4 mAb, anti-CD69 mAb, and anti-CD86 mAb. Dead cells were stained with PI. The cells were analyzed by flow cytometry. (A–C) Viability of total spleen cells (A), of B220^+^ cells (B), and of CD4^+^ cells (C) cultured without *T*. *halophilus* as a control was defined as 100%. Based on this parameter, the relative viability of cells cultured with *T*. *halophilus* were calculated. Bars indicate mean ± S.D (n = 8). (D, E) The CD86^+^ cells of B220^+^ cells and CD69^+^ cells of CD4^+^ cells cultured without *T*. *halophilus* as a control were defined as 100%. Based on this aspect, the relative CD86^+^ cell proportion of B220^+^ cells (D) and CD69^+^ cells of CD4^+^ cells (E) cultured with *T*. *halophilus* were calculated. Bars indicate mean ± S.D (n = 8). *p < 0.05 and **p < 0.01 to control *t*-test, respectively.

We isolated several strains of a halophilic LAB, *T*. *halophilus*, from miso. To identify the properties of the isolates in the aspect of a healthy diet, we evaluated the stimulatory function of the isolates on immune cells. Initially, we established our own *in vitro* immunological assay by using B cells from the mouse spleen based on activation markers such as CD86 on B cells and their viability. We selected 56 strains and tested their effect on immune cell stimulation. We shortlisted 7 isolates (Nos. 1, 3, 13, 15, 19, 30, and 31), which increased both the CD86 expression and viability of B cells (Fig 1). However, other strains did not increase either the viability or the CD86 expression. Next, we further evaluated the immunostimulatory effects of these strains on not only B cells but also T cells together with those of control strains. As shown in Fig 2, we examined these strains based on the activation markers such as CD86 on B cells and dendritic cells and as CD69 on T cells and their viability. Except for No. 3 isolate, all isolates augmented viability of splenocytes, including T and B cells (Fig 2A–2C). Isolate Nos. 1, 3, and 31 significantly increased the CD86 expression on B cells. On the other hand, all isolates increased the CD69 expression on CD4 T cells. Overall, isolate No. 1 appeared to be the most effective strain of immunomodulatory activity. In addition, based on the partial 16s ribosomal RNA sequence, all of these strains were different from the one in the BLAST database (S1 Fig), indicating that these are novel strains.

### *T*. *halophilus* No. 1 alters the gene expression profile in mouse B cells

**Fig 3:**
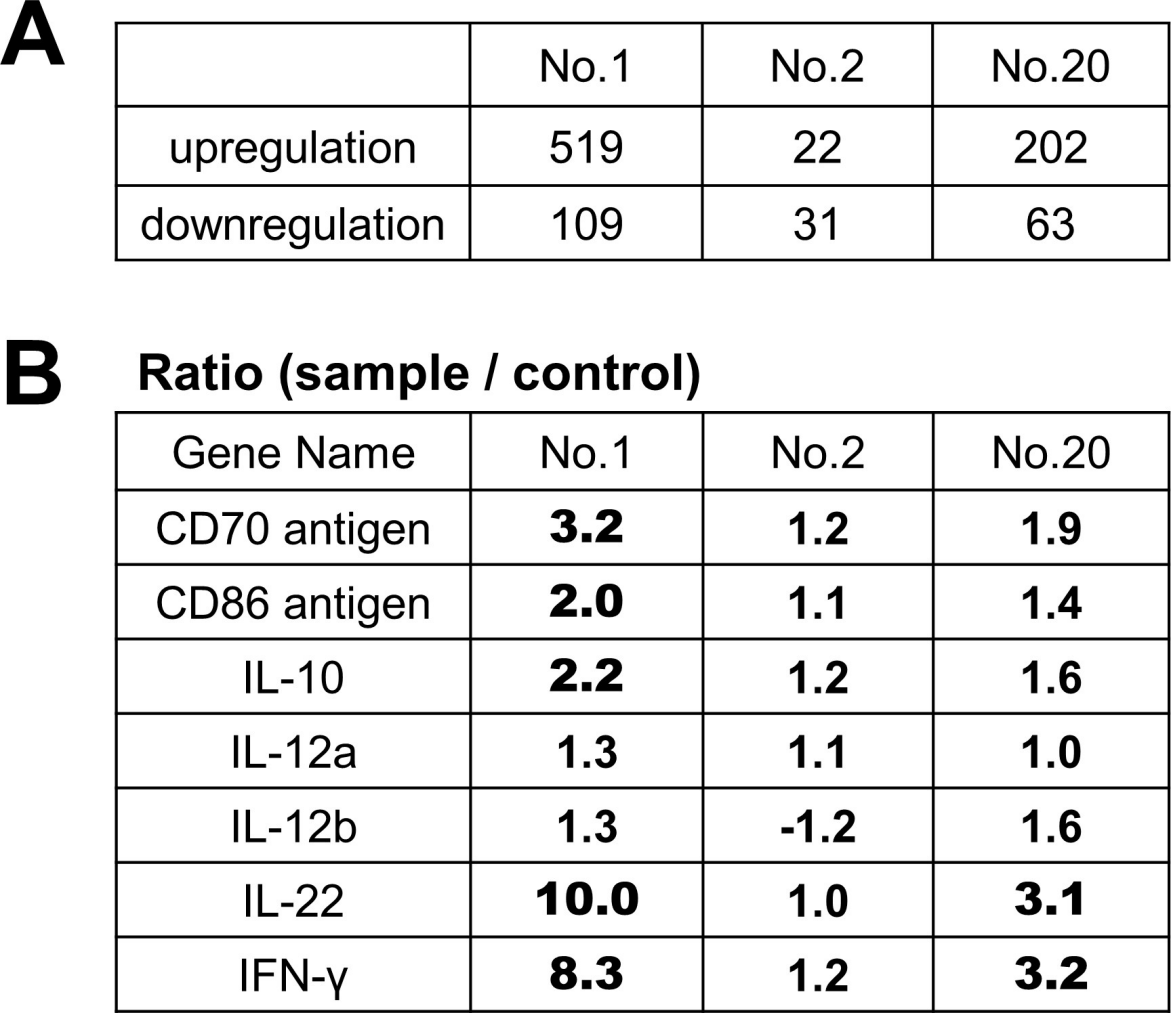
The gene expression profile of B cells cultured with *T*. *halophilus*. The spleen cells from C57BL/6 mice were cultured with each indicated strain, and B220^+^ cells were isolated. After total RNA preparation, the samples were subjected to DNA microarray analyses. The gene numbers significantly increased and decreased their expression upon each of *T*. *halophilus* strain are shown (A). The representative results of the genes related to the immune systems are shown (B). The ratio over 2 indicates significant changes.

To clarify the gene expression profile mediated by *T*. *halophilus*, we further analyzed isolate No. 1, which showed the most efficient activity together with isolate No. 2 and 20 as controls by DNA microarray. Among more than 55,000 genes, approximately 1,000 genes were identified to be either induced or suppressed by *T*. *halophilus* No. 1 in B cells, while No. 2 and No. 20 exhibited less influences (Fig 3A). The addition of isolate No. 1 in the culture medium augmented the gene expression of CD86 and CD70—which are co-stimulatory molecules [29, 30] known to interact with CD28 and CD27 on T cells (Fig 3B)—suggesting that isolate No. 1 facilitated immune responses. Thus, the increase in the CD86 gene expression is consistent with the result given in Fig 1. Furthermore, isolate No. 1 augmented the levels of cytokines such as IL-10 and INF-γ. Especially, we found that *T*. *halophilus* No.1 mediated IL-22 induction in B cells for the first time. On the other hand, IL-12 or IFN-β which are known to be augmented by LAB [8, 31, 32], were not increased by the addition of isolate No. 1.

### *T*. *halophilus* No. 1 increased the IL-22, IL-10, and IFN-γ production in B cells from the mouse spleen

**Fig 4:**
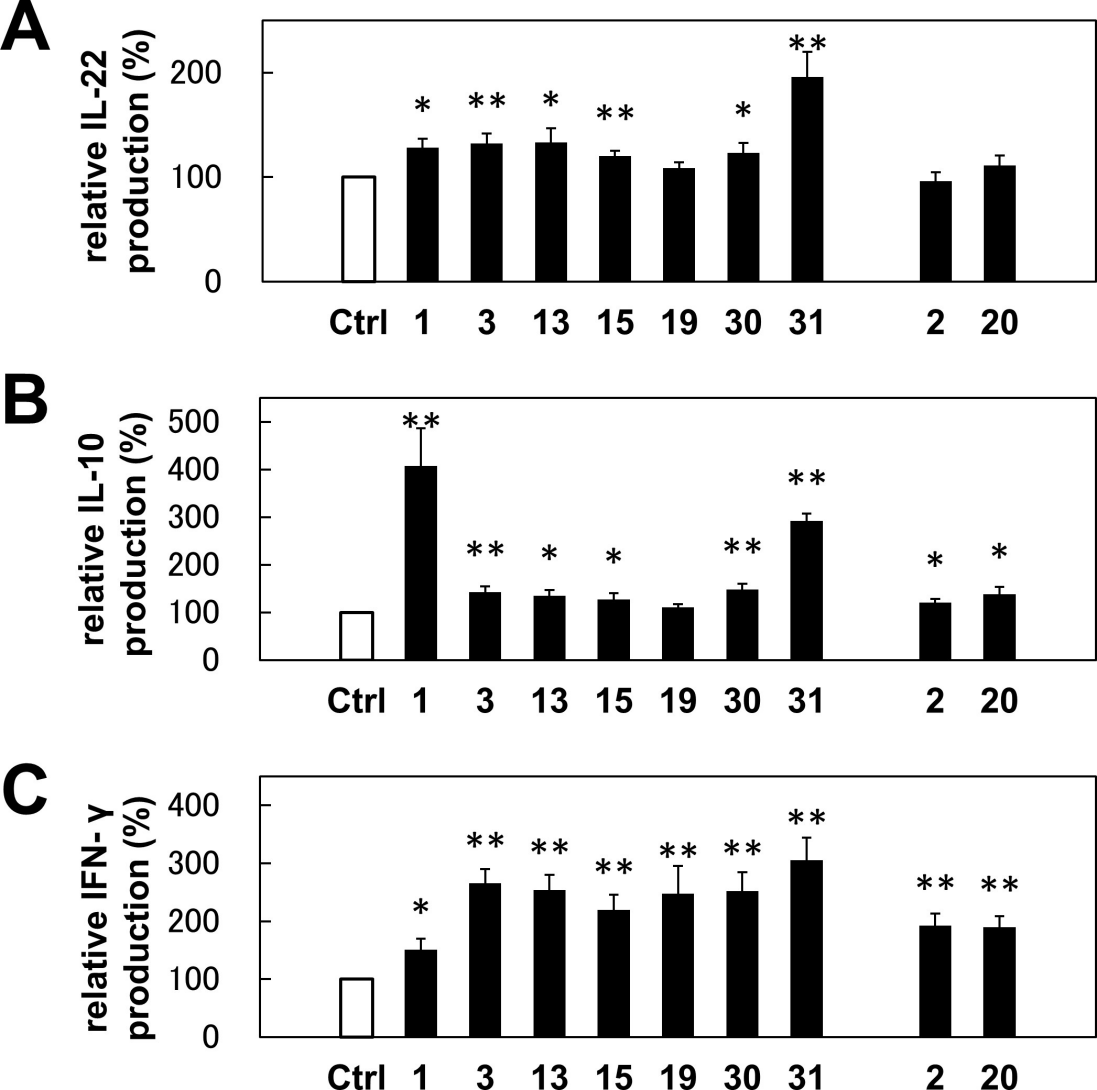
Cytokine production in B cells cultured with *T*. *halophilus*. The spleen cells from C57BL/6 mice were cultured with 10 μg of *T*. *halophilus* strains in 1 mL of PRMI1640 medium containing 10% FCS for 2 days. The cells were further incubated with GolgiStop and then collected and treated with BD Fixation/Permeabilization Solution Kit. Subsequently, the cells were stained and analyzed by flow cytometry. Each cytokine positive cells, IL-22 (A), IL-10 (B), and IFN-γ (C) of B220^+^ cells cultured without *T*. *halophilus* as a control was defined as 100%. Based on these aspects, the relative cytokine positive cells cultured with *T*. *halophilus* were calculated. Bars indicate mean ± S.D (n = 6). *p < 0.05 and **p < 0.01 to control of *t*-test, respectively.

**Fig 5:**
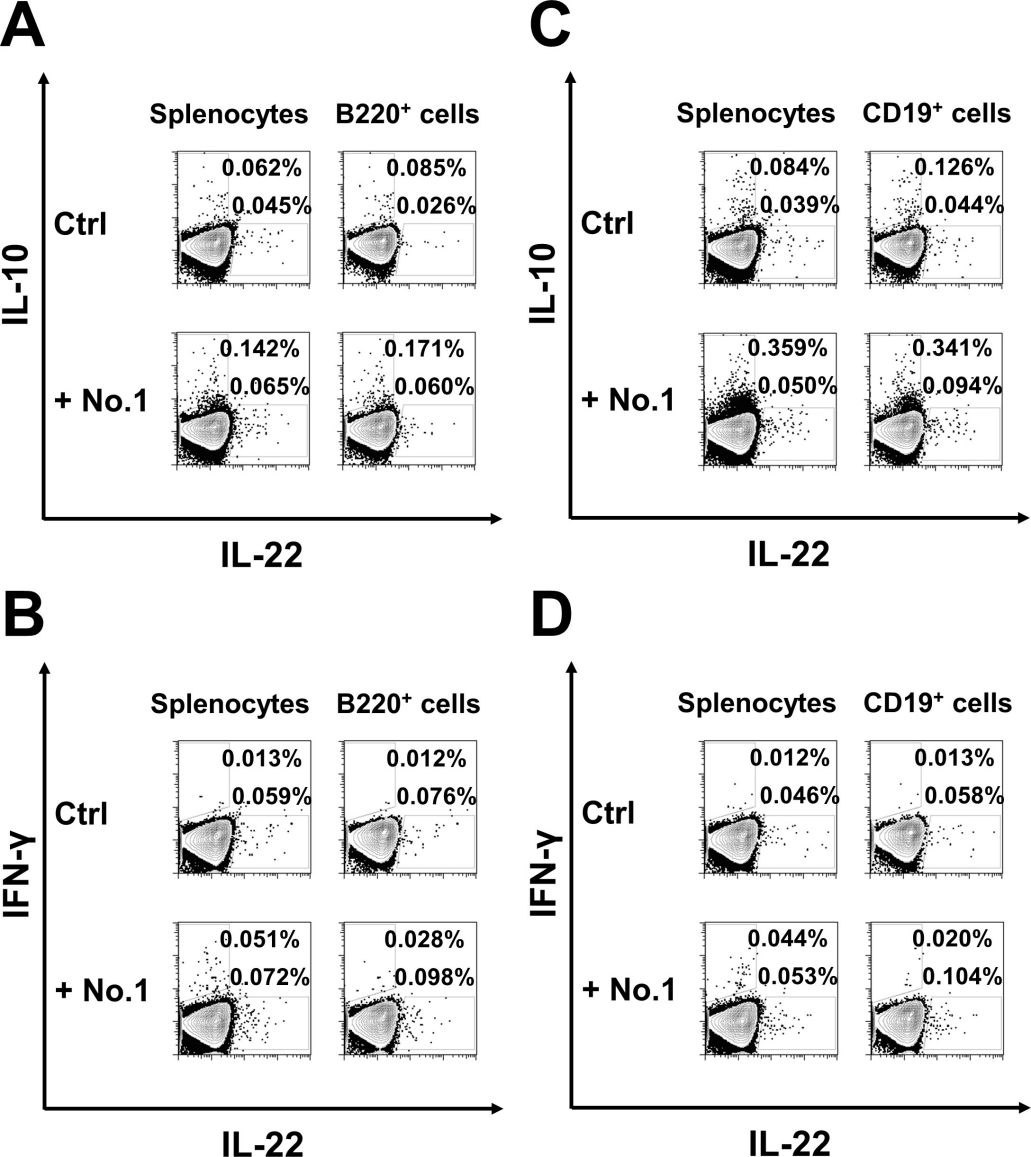
The profile of cytokine production induced by *T*. *halophilus* strains No.1. An example of the measurement of flow cytometry in Fig 4 is indicated. The spleen cells from C57BL/6 mice were cultured with or without 10 μg of *T*. *halophilus* strains No. 1 in 1 mL of PRMI1640 medium containing 10% FCS for 2 days. IL-10 and IL-22 of B220^+^ cells (A), IFN-γ and IL-22 of B220^+^ cells (B), IL-10 and IL-22 of CD19^+^ cells (C), IFN-γ and IL-22 of CD19^+^ cells (D).

**Fig 6:**
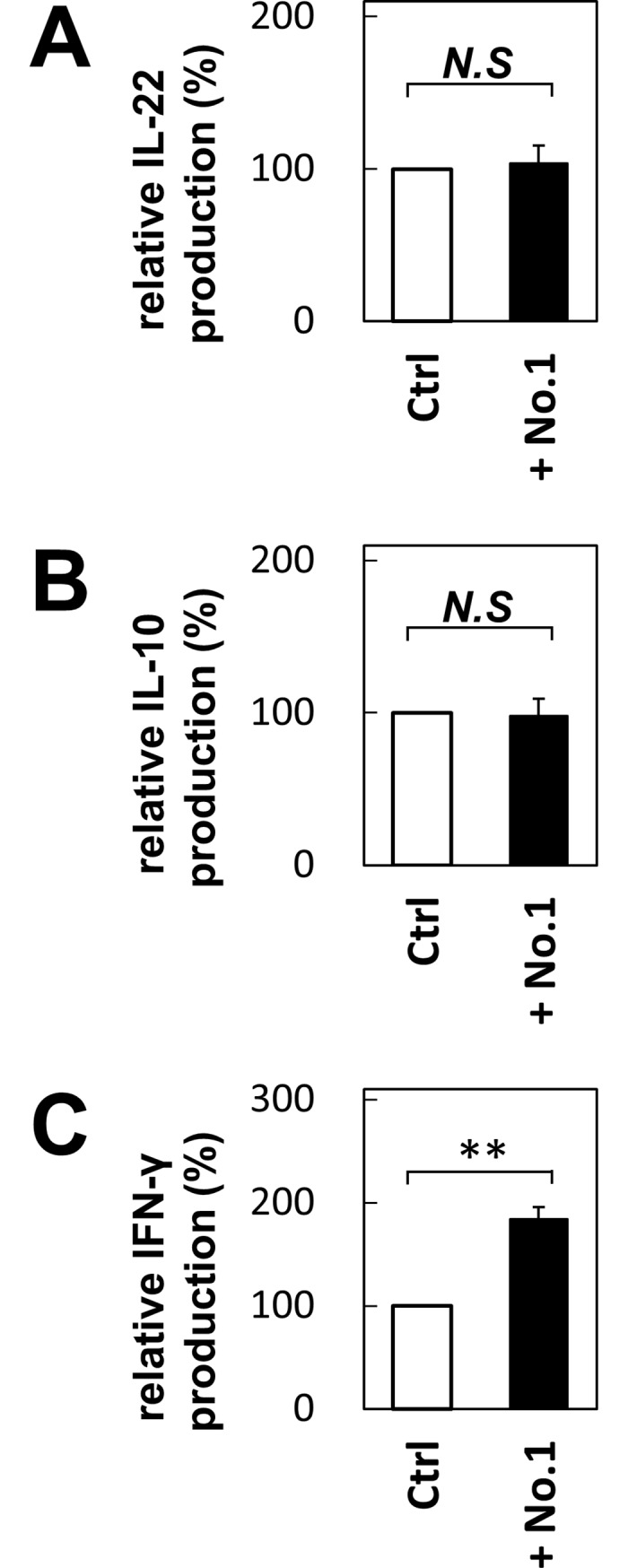
Cytokine production in T cells cultured with *T*. *halophilus* strains No.1. The spleen cells from C57BL/6 mice were cultured with 10 μg of *T*. *halophilus* strains No.1 in 1 mL of PRMI1640 medium containing 10% FCS for 2 days. The cells were further incubated with GolgiStop and then collected and treated with BD Fixation/Permeabilization Solution Kit. Subsequently, the cells were stained and analyzed by flow cytometry. Each cytokine positive cells, IL-22 (A), IL-10 (B), and IFN-γ (C) of CD4^+^ cells cultured without *T*. *halophilus* as a control was defined as 100%. Based on these aspects, the relative cytokine positive cells cultured with *T*. *halophilus* were calculated. Bars indicate mean ± S.D. (n = 8). **p < 0.01 to control of *t*-test.

As DNA microarray analyses revealed, *T*. *halophilus* No. 1 mediated the induction of IL-22, IL-10, and IFN-γ at the mRNA level. We next examined their production at the protein level with the candidate isolate Nos. 1, 3, 13, 15, 19, 30, and 31 together with others as controls (Fig 4). As shown in Fig 4A, all these strains, except for No. 19, augmented the IL-22 production in B cells. As compared with other candidates, No. 1 strains augmented the IL-10 production, whereas it is less-effective for INF-γ induction (Fig 4B and 4C). Isolate No. 31 increased both IL-10 and INF-γ production. Other isolates facilitated the salient IFN-γ induction, although they did not induce IL-10 production effectively. According to the FACS profiles, we found that the subpopulations of B cells produced these cytokines exclusively (Fig 5A and 5B). We found that almost no double producer cells exist. Furthermore, to exclude the possibility that the minor population of B220^+^ cells, plasmacytoid dendritic cells, were not the main cytokine producers of B220^+^ cells, we also examined the CD19^+^ cells. As shown in Fig 5C and 5D, we also confirmed B cell-mediated cytokine productions. We further examined the cytokine production in T cells. Upon addition of *T*. *halophilus* No. 1, IFN-γ was increased in the CD4^+^ T cells though IL-10 and IL-22 were not altered (Fig 6). *T*. *halophilus* No. 1 affected cytokine productions in both B cells and T cells, though their cytokine profiles were different.

### *T*. *halophilus* No. 1 increased the IgA and IgG production in spleen B cells

**Fig 7:**
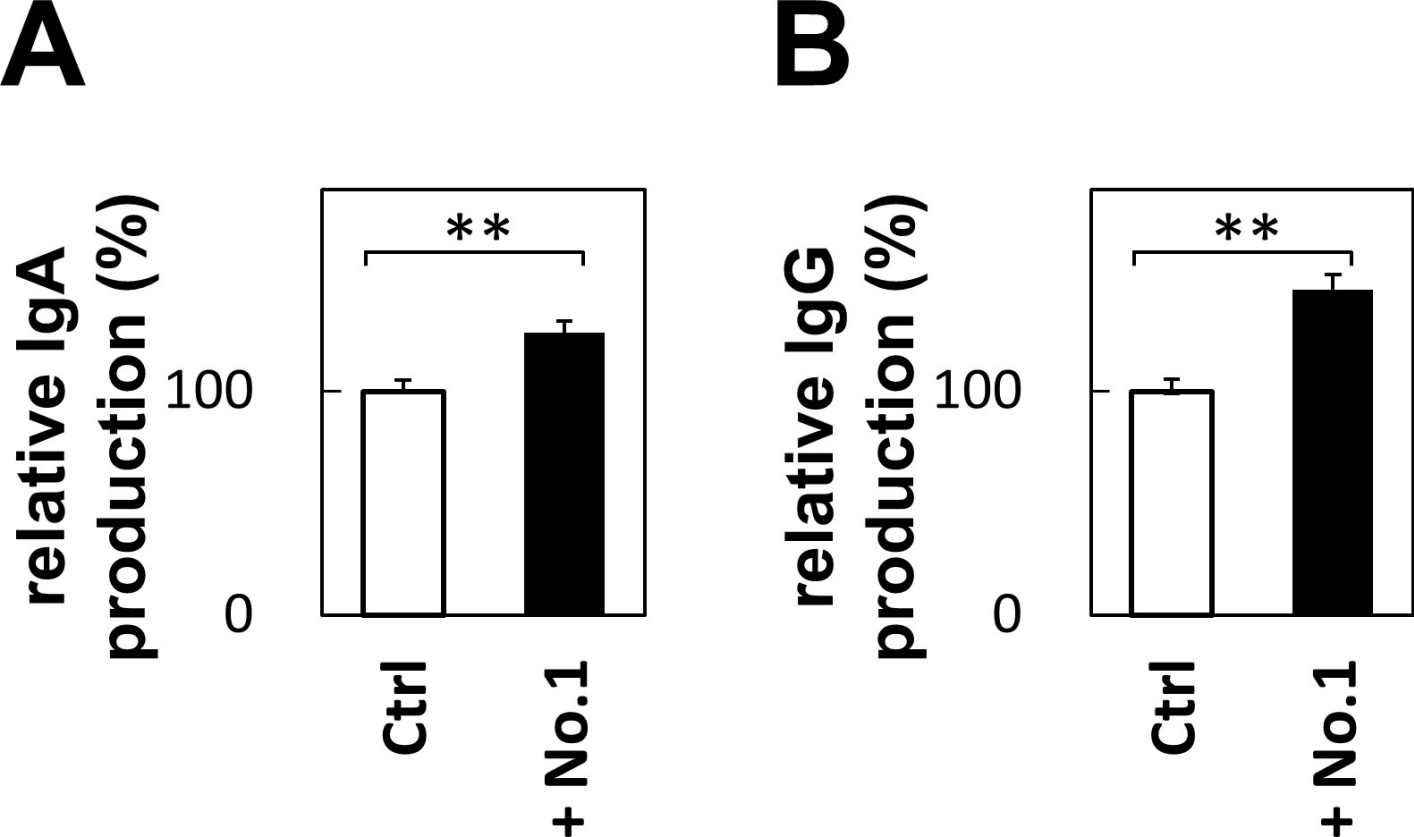
IgA and IgG production cultured with *T*. *halophilus in vitro*. The spleen cells from C57BL/6 mice were cultured with 10 μg of *T*. *halophilus* strains No.1 in 1 mL of PRMI1640 medium containing 10% FCS for 2 days. Then, the cell culture supernatant samples were obtained, and the IgA (A) and IgG (B) levels were analyzed by ELISA. Bars indicate mean ± S.D (n = 6). **p < 0.01 to control of *t*-test.

As *T*. *halophilus* No.1 increased the cytokine productions in spleen B cells, we further examined whether it increase Ig productions or not. We cultured spleen cells with *T*. *halophilus* No. 1, and measured Ig production in the culture supernatants. As shown in Fig 7, IgA and IgG productions were increased upon the presence of *T*. *halophilus* No. 1. This strongly suggests that *T*. *halophilus* No. 1 augments Ig productions in B cells directly.

### *T*. *halophilus* No.1-containing diet increased the serum IgA level in C57BL/6 mice

**Fig 8:**
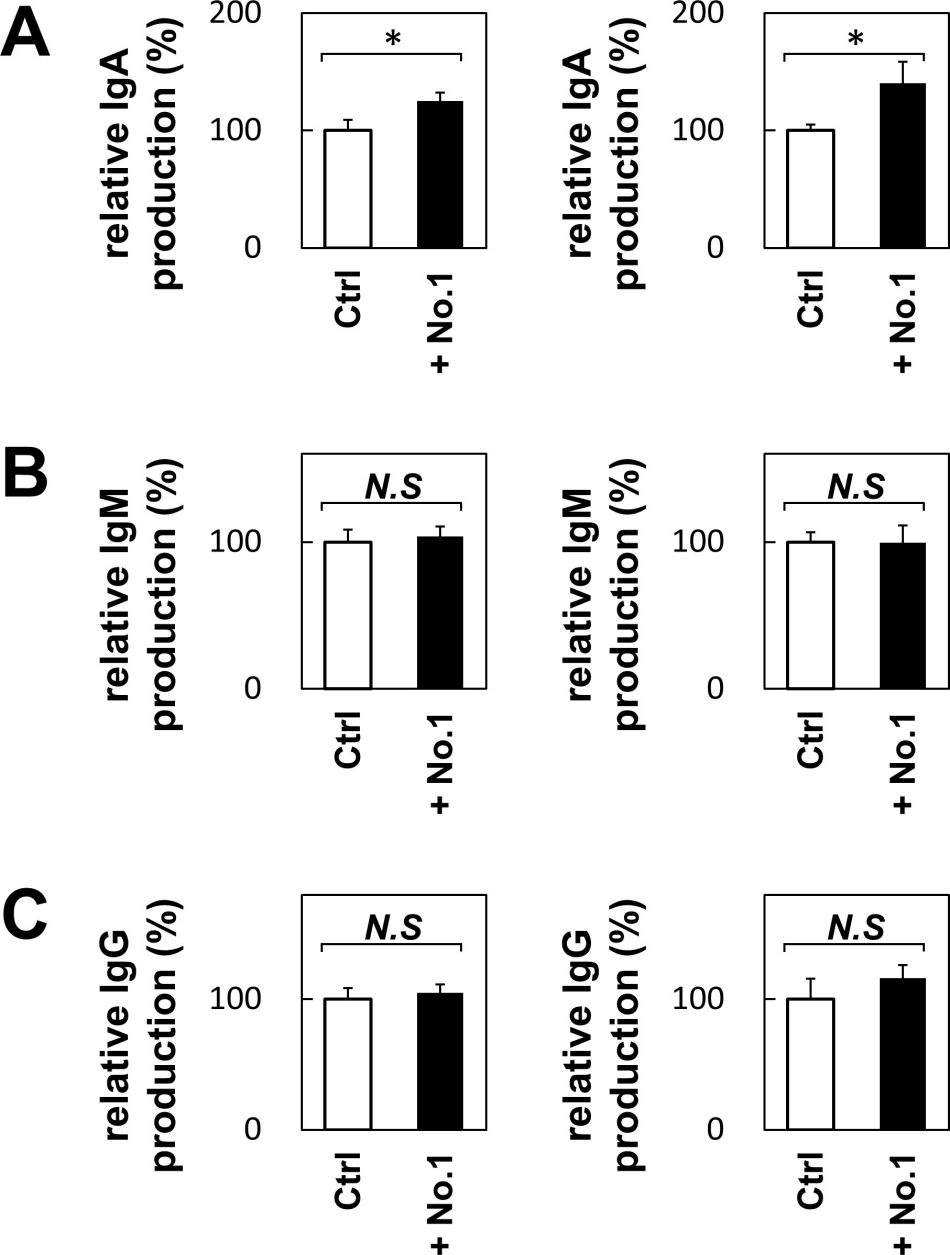
The effect of *T*. *halophilus* on the serum IgA, IgM, and IgG in mice. Diet containing 1% *T*. *halophilus* were fed to C57BL/6 mice for 2 weeks. Then, the serum samples were obtained, and the serum IgA, IgM, and IgG levels were analyzed by ELISA. Mice fed without *T*. *halophilus* were used as control. Bars indicate mean ± S.D. (n = 3 mice). The results of two experiments are shown. *p < 0.05 and **p < 0.01 to control *t*-test, respectively.

**Fig 9:**
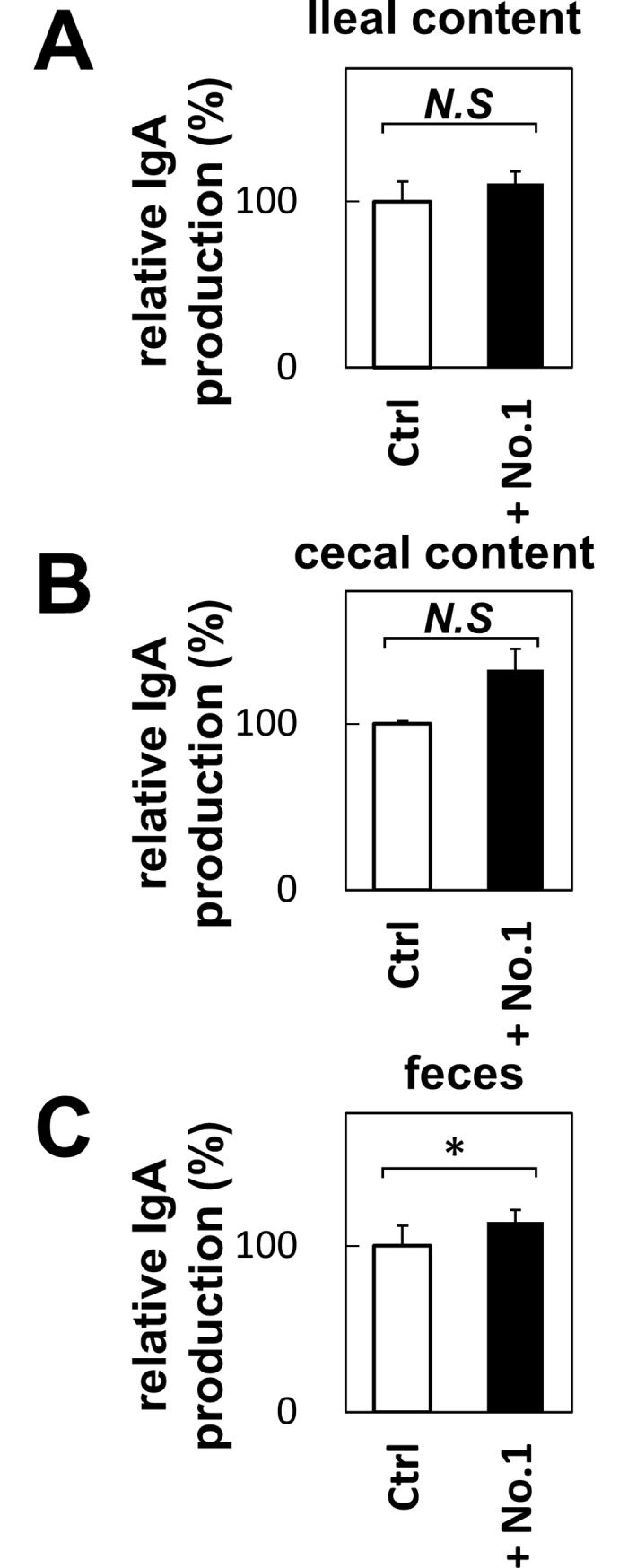
The effect of *T*. *halophilus* on IgA production in the feces and ileal and cecal contents in mice. Diet containing 1% *T*. *halophilus* were fed to C57BL/6 mice for 2 weeks. Then, the feces and the ileum and the cecum were obtained. The feces were suspended in 10-fold of PBS (w/v), and used abstraction liquid for measurement of ELISA. Contents of the ileum and the cecum were suspended in 5-fold and 3-fold of PBS (w/v), respectively, and their supernatant were subjected to ELISA. Mice fed without *T*. *halophilus* were used as control. Bars indicate mean ± S.D. of contents of the ileum (A), contents of the cecum (B), and feces (C) (n = 3 mice). *p < 0.05 to control *t*-test.

We showed that *T*. *halophilus* demonstrates immunomodulatory activity *in vitro*. Next, we examined their biological activity *in vivo*. For this purpose, we fed 1% *T*. *halophilus* No. 1-containing diet to the experimental mice for 2 weeks and then analyzed the surface markers of splenocytes and serum immunoglobulin M (IgM), IgG, and IgA. Although *T*. *halophilus*-containing diet did not alter the CD86 expression on B cells, the serum IgA level was significantly increased (Fig 8). It did not change the serum IgM and IgG levels significantly. We further examined the IgA level in the ileum, cecum and feces. Although IgA level in the ileal content was not altered, that in the feces was significantly increased upon *T*. *halophilus* No. 1. feeding (Fig 9). The IgA level in the cecal content tends to be increased. Thus, the feeding of *T*. *halophilus* No. 1 augmented the IgA production, demonstrating immunomodulatory functions *in vivo*.

### *T*. *halophilus* No. 1-containing diet augmented OVA-specific immune responses in C57BL/6 mice

**Fig 10:**
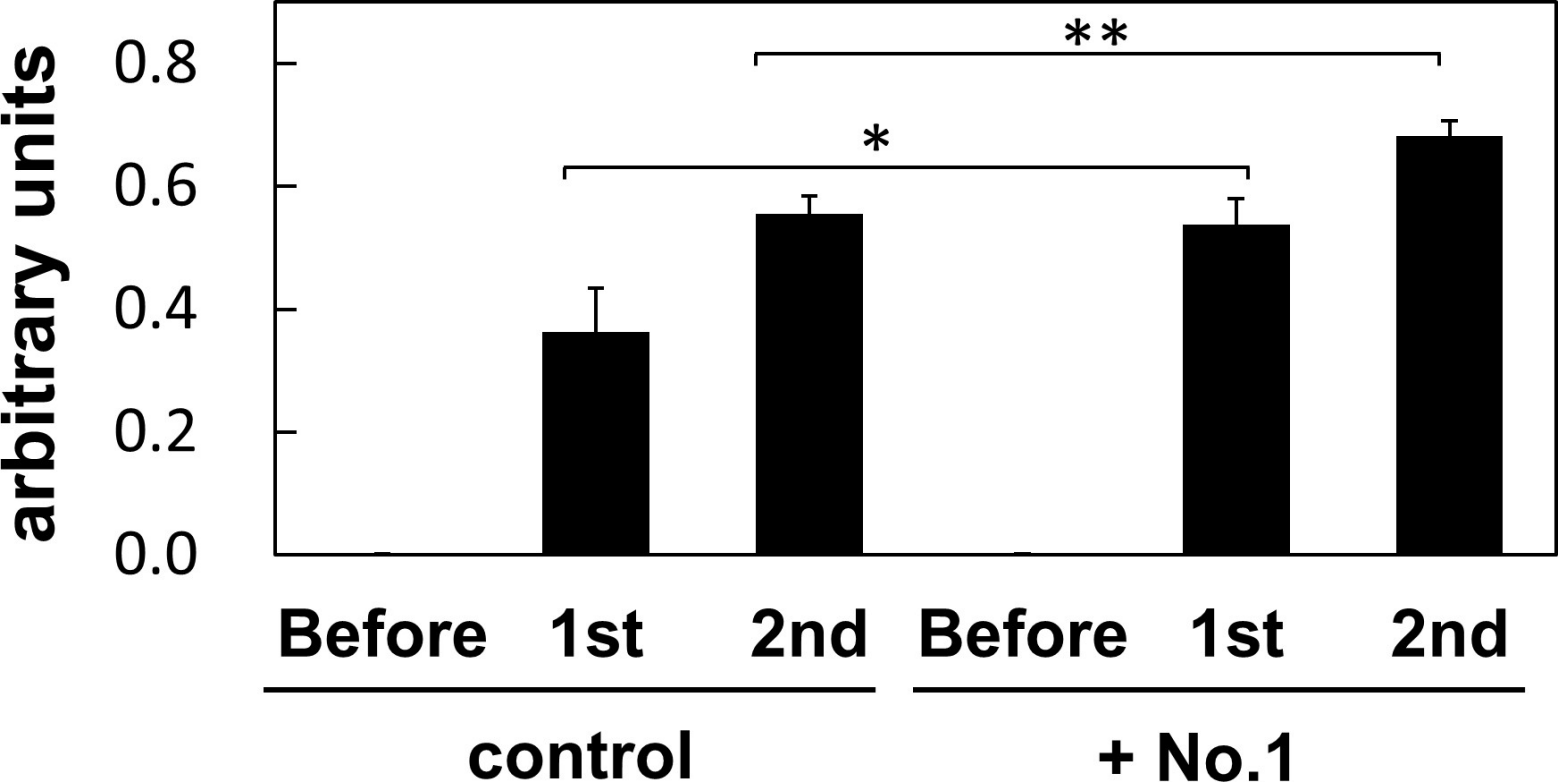
The effect of *T*. *halophilus* on antigen-specific IgG response in mice. Diet containing 1% *T*. *halophilus* were fed to C57BL/6 mice for 2 weeks. Then, the mice were immunized, and, after 4 weeks, a booster with OVA/alum was applied intraperitoneally. The serum samples obtained at the indicated time points and OVA-specific IgG were analyzed by ELISA. Mice fed without *T*. *halophilus* served as controls. Bars indicate mean ± S.D. (n = 3 mice). *p < 0.05 and **p < 0.01 to control *t*-test, respectively.

*T*. *halophilus* No. 1-feeding mice augmented the IgA production *in vivo*. We examined this strains’ effect on immune responses. After 2 weeks of *T*. *halophilus* No. 1-feeding, we immunized C57BL/6 mice with OVA/alam intraperitoneally and applied a booster with the same antigen after 4 weeks. As shown in Fig 10, *T*. *halophilus* No. 1-feeding mice significantly produced more OVA-specific IgG in the serum than the control mice. Secondary immune responses were also augmented upon *T*. *halophilus* No. 1 strain administration. These results indicate that *T*. *halophilus* No. 1 possesses immunostimulatory activity and elevates the antigen-specific IgG level.

## Discussion

In this study, we screened *T*. *halophilus* strains from miso for their immunomodulatory functions and identified 7 *T*. *halophilus* strains (Nos. 1, 3, 13, 15, 19, 30, and 31) as potential probiotics. These strains preferentially induced an activation marker CD86 on B cells and cytokines. In addition, we found that *T*. *halophilus* No. 1 induced IL-22 cytokine production in B cells for the first time. Furthermore, the feeding of *T*. *halophilus* No. 1 strain augmented the amount of total serum IgA and antigen-specific serum IgG responses in mice. Thus, we identified the novel immunomodulatory functions of *T*. *halophilus* No. 1 *in vivo*.

Miso has been indicated as a healthy fermented food. Here we showed that miso-derived *T*. *halophilus* possesses immunomodulatory activities, including a novel function of IL-22 induction in B cells and INF-γ induction in T cells. Until date, *T*. *halophilus* has been shown to regulate immune cells such as dendritic cells and T cells. A *T*. *halophilus* MN45 strain, which is isolated from miso, augments INF-γ and IL-12 production and reduces IgE production [19], resulting in the alleviation of atopic allergy in mice. Furthermore, *T*. *halophilus* Th221 isolated from soy sauce has been shown to repress the serum IgE level and control allergic rhinitis in human [33]. *T*. *halophilus* strain KK221 strain induces INF-β through TLR3 and TLR9 on dendritic cells and contributes to the anti-inflammatory function against inflammatory bowel diseases [26]. Thus, in consensus with previous studies, our study also strongly suggests *T*. *halophilus* as beneficial bacteria for our health.

In this study, we identified a novel cytokine-producing B cell subset, although B cells have been reported to produce cytokines such IL-2, IL-4, IL-6, IL-10, IL-17, and IFN-γ [34–39]. Among these cells, the IL-10-producing B cell subset is known as a regulatory B cell subset [40, 41]. IL-22 is a member of the IL-10 superfamily and contributes to the protection of the mucosal barriers against microbial parasites in the skin, lung, and intestine [42, 43]. Activated NK and T cells, LTi and ILC3, have been shown to produce IL-22 [44] [45]. IL-22 is involved in the initiation of innate immune responses against pathogens in the gut [46]. As B cells are abundant in the gut tissue, IL-22-producing B cell subset also appears to contribute to the immune responses in the gut along with IL-22-producing T cells and ILC3.

We identified the expressions of several genes in *T*. *halophilus* No. 1-treated B cells. In addition to IL-22, the activation markers such as CD86 and CD70 were upregulated upon *T*. *halophilus* No. 1 administration, as analyzed by flow cytometry. Since these co-stimulatory molecules are known to regulate immune responses [29, 30], *T*. *halophilus* No. 1 seems to possess immunomodulatory activity.

We showed that *T*. *halophilus* No. 1-feeding mice augmented the serum IgA level. Previously, bacterial cells and polysaccharides of *Leuconostoc mesenteroides* strain NTM048 and strain JCM6124(T) induced the IgA production in Peyer’s patch cells [47]. Furthermore, the oral administration of *L*. *mesenteroides* strain NTM048 increased the fecal IgA content in mice [48]. Recently, the production of cytokines such as IL-6 and IL-10 from dendritic cells has been suggested to augment the IgA production [49]. Indeed, the oral administration of *T*. *halophilus* No. 1 increased the IL-10 production in the spleen cells (Fig 4B). Furthermore, *T*. *halophilus* No. 1 increased the IgA production in the spleen cells, suggesting the direct effect. It is also possible to account for the T cell-mediated effect on IgA production. Although IL-5, IL-6 IL-10 and TGF-β are important for IgA production, T cell-mediated effect of them has not been reported so far. We also presented that *T*. *halophilus* did not induce IL-10 in T cells (Fig 6). Based on these evidences, *T*. *halophilus* No. 1 may contribute to increase the serum IgA level.

We showed that *T*. *halophilus* No. 1 augmented the IgG immune responses against OVA, suggesting that this strain possesses strong immunostimulatory activity. In concordance with our results, the oral administration of *Lactobacillus* GG significantly augmented the antigen-specific serum IgG in a previous study [50]. Furthermore, antigen-specific IgA and IL-6 were elevated in *Lactobacillus* GG-fed mice. These results indicate that some species of LAB possess immunostimulatory activity.

Cumulatively, we identified several strains of *T*. *halophilus* isolated from miso as probiotics, although other constituents of miso may also account for its beneficial effect on our health. To clarify the molecular mechanisms of individual components of miso, its constituents need to be studied in the future.

## Supporting information

S1 Fig Comparison of the DNA sequences for partial 16s RNA of *T*. *halophilus*. The partial DNA sequences for the *T*. *halophilus* (Nos. 1, 3, 13, 15, 19, 30, and 31) 16s RNA were shown and compared with the most homologous sequence in the blast database (*Tetragenococcus halophilus* subsp. halophilus strain IAM 1676 16S ribosomal RNA, partial sequence: NR_122102). The differences were indicated by red.
(DOCX)
