# Supplementary material for: Isolation of immune-regulatory Tetragenococcus halophilus from miso
Source: PLoS One. 2018 Dec 26;13(12):e0208821. doi: 10.1371/journal.pone.0208821 (PMC6306251; doi:10.1371/journal.pone.0208821)
Supplement: S1 Fig — The partial DNA sequences for the T. halophilus (Nos. 1, 3, 13, 15, 19, 30, and 31) 16s RNA were shown and compared with the most homologous sequence in the blast database (Tetragenococcus halophilus subsp. halophilus strain IAM 1676 16S ribosomal RNA, partial sequence: NR_122102). The differences were indicated by red. (DOCX) [file pone.0208821.s001.docx]

IAM1676 32 ATACATGCAAGTCGAACGCTGCTTAAGAAGAAACTTCGGTTTTTTCTTAAGCGGAGTGGCGGACGGGTGAGTAACACGTG 111

No.1 1 ATACATGCAAGTCGAACGCTGCTTAAGAAGAAACTTCGGTTTTTTCTTAAGCGGAGTGGCGGACGGGTGAGTAACACGTG 80

No.3 1 ATACATGCAAGTCGAACGCTGCTTAAGAAGAAACTTCGGTTTTTTCTTAAGCGGAGTGGCGGACGGGTGAGTAACACGTG 80

No.13 1 ATACATGCAAGTCGAACGCTGCTTAAGAAGAAACTTCGGTTTTTTCTTAAGCGGAGTGGCGGACGGGTGAGTAACACGTG 80

No.15 1 ATACATGCAAGTCGAACGCTGCTTAAGAAGAAACTTCGGTTTTTTCTTAAGCGGAGTGGCGGACGGGTGAGTAACACGTG 80

No.19 1 ATACATGCAAGTCGAACGCTGCTTAAGAAGAAACTTCGGTTTTTTCTTAAGCGGAGTGGCGGACGGGTGAGTAACACGTG 80

No.30 1 ATACATGCAAGTCGAACGCTGCTTAAGAAGAAACTTCGGTTTTTTCTTAAGCGGAGTGGCGGACGGGTGAGTAACACGTG 80

No.31 1 ATACATGCAAGTCGAACGCTGCTTAAGAAGAAACTTCGGTTTTTTCTTAAGCGGAGTGGCGGACGGGTGAGTAACACGTG 80

IAM1676 112 GGGAACCTATCCATCAGCGGGGGATAACACTTGGAAACAGGTGCTAATACCGCATA**T**GGCTTTTTTTCACCTGAAAGAAA 191

No.1 81 GGGAACCTATCCATCAGCGGGGGATAACACTTGGAAACAGGTGCTAATACCGCATA**T**GGCTTTTTTTCACCTGAAAGAAA 160

No.3 81 GGGAACCTATCCATCAGCGGGGGATAACACTTGGAAACAGGTGCTAATACCGCATA**C**GGCTTTTTTTCACCTGAAAGAAA 160

No.13 81 GGGAACCTATCCATCAGCGGGGGATAACACTTGGAAACAGGTGCTAATACCGCATA**T**GGCTTTTTTTCACCTGAAAGAAA 160

No.15 81 GGGAACCTATCCATCAGCGGGGGATAACACTTGGAAACAGGTGCTAATACCGCATA**C**GGCTTTTTTTCACCTGAAAGAAA 160

No.19 81 GGGAACCTATCCATCAGCGGGGGATAACACTTGGAAACAGGTGCTAATACCGCATA**T**GGCTTTTTTTCACCTGAAAGAAA 160

No.30 81 GGGAACCTATCCATCAGCGGGGGATAACACTTGGAAACAGGTGCTAATACCGCATA**T**GGCTTTTTTTCACCTGAAAGAAA 160

No.31 81 GGGAACCTATCCATCAGCGGGGGATAACACTTGGAAACAGGTGCTAATACCGCATA**T**GGCTTTTTTTCACCTGAAAGAAA 160

IAM1676 192 GCTCAAAGGCGCTTTACAGCGTCACTGATGGCTGG**T**CCCGCGGTGCATTAGCCAGTTGGTGAGGTAACGGCTCACCAAAG 271

No.1 161 GCTCAAAGGCGCTTTACAGCGTCACTGATGGCTGG**T**CCCGCGGTGCATTAGCCAGTTGGTGAGGTAACGGCTCACCAAAG 240

No.3 161 GCTCAAAGGCGCTTTACAGCGTCACTGATGGCTGG**C**CCCGCGGTGCATTAGCCAGTTGGTGAGGTAACGGCTCACCAAAG 240

No.13 161 GCTCAAAGGCGCTTTACAGCGTCACTGATGGCTGG**T**CCCGCGGTGCATTAGCCAGTTGGTGAGGTAACGGCTCACCAAAG 240

No.15 161 GCTCAAAGGCGCTTTACAGCGTCACTGATGGCTGG**C**CCCGCGGTGCATTAGCCAGTTGGTGAGGTAACGGCTCACCAAAG 240

No.19 161 GCTCAAAGGCGCTTTACAGCGTCACTGATGGCTGG**T**CCCGCGGTGCATTAGCCAGTTGGTGAGGTAACGGCTCACCAAAG 240

No.30 161 GCTCAAAGGCGCTTTACAGCGTCACTGATGGCTGG**T**CCCGCGGTGCATTAGCCAGTTGGTGAGGTAACGGCTCACCAAAG 240

No.31 161 GCTCAAAGGCGCTTTACAGCGTCACTGATGGCTGG**T**CCCGCGGTGCATTAGCCAGTTGGTGAGGTAACGGCTCACCAAAG 240

IAM1676 272 CAACGATGCATAGCCGACCTGAGAGGGTGATCGGCCACACTGGGACTGAGACACGGCCCAAACTCCTACGGGAGGCAGCA 351

No.1 241 CAACGATGCATAGCCGACCTGAGAGGGTGATCGGCCACACTGGGACTGAGACACGGCCCAAACTCCTACGGGAGGCAGCA 320

No.3 241 CAACGATGCATAGCCGACCTGAGAGGGTGATCGGCCACACTGGGACTGAGACACGGCCCAAACTCCTACGGGAGGCAGCA 320

No.13 241 CAACGATGCATAGCCGACCTGAGAGGGTGATCGGCCACACTGGGACTGAGACACGGCCCAAACTCCTACGGGAGGCAGCA 320

No.15 241 CAACGATGCATAGCCGACCTGAGAGGGTGATCGGCCACACTGGGACTGAGACACGGCCCAAACTCCTACGGGAGGCAGCA 320

No.19 241 CAACGATGCATAGCCGACCTGAGAGGGTGATCGGCCACACTGGGACTGAGACACGGCCCAAACTCCTACGGGAGGCAGCA 320

No.30 241 CAACGATGCATAGCCGACCTGAGAGGGTGATCGGCCACACTGGGACTGAGACACGGCCCAAACTCCTACGGGAGGCAGCA 320

No.31 241 CAACGATGCATAGCCGACCTGAGAGGGTGATCGGCCACACTGGGACTGAGACACGGCCCAAACTCCTACGGGAGGCAGCA 320

IAM1676 352 GTAGGGAATCTTCGGCAATGGACGCAAGTCTGACCGAGCAACGCCGCGTGAGTGAAGAAGGTTTTCGGATCGTAAAGCTC 431

No.1 321 GTAGGGAATCTTCGGCAATGGACGCAAGTCTGACCGAGCAACGCCGCGTGAGTGAAGAAGGTTTTCGGATCGTAAAGCTC 400

No.3 321 GTAGGGAATCTTCGGCAATGGACGCAAGTCTGACCGAGCAACGCCGCGTGAGTGAAGAAGGTTTTCGGATCGTAAAGCTC 400

No.13 321 GTAGGGAATCTTCGGCAATGGACGCAAGTCTGACCGAGCAACGCCGCGTGAGTGAAGAAGGTTTTCGGATCGTAAAGCTC 400

No.15 321 GTAGGGAATCTTCGGCAATGGACGCAAGTCTGACCGAGCAACGCCGCGTGAGTGAAGAAGGTTTTCGGATCGTAAAGCTC 400

No.19 321 GTAGGGAATCTTCGGCAATGGACGCAAGTCTGACCGAGCAACGCCGCGTGAGTGAAGAAGGTTTTCGGATCGTAAAGCTC 400

No.30 321 GTAGGGAATCTTCGGCAATGGACGCAAGTCTGACCGAGCAACGCCGCGTGAGTGAAGAAGGTTTTCGGATCGTAAAGCTC 400

No.31 321 GTAGGGAATCTTCGGCAATGGACGCAAGTCTGACCGAGCAACGCCGCGTGAGTGAAGAAGGTTTTCGGATCGTAAAGCTC 400

IAM1676 432 TGTTGTCAGCAAAGAACAGGAGAAAGAGGAAATGCTTTTTC**C**ATGACGGTAGCTGACCAGAAAGCCACGGCTAACTACGT 511

No.1 401 TGTTGTCAGCAAAGAACAGGAGAAAGAGGAAATGCTTTTTC**T**ATGACGGTAGCTGACCAGAAAGCCACGGCTAACTACGT 480

No.3 401 TGTTGTCAGCAAAGAACAGGAGAAAGAGGAAATGCTTTTTC**T**ATGACGGTAGCTGACCAGAAAGCCACGGCTAACTACGT 480

No.13 401 TGTTGTCAGCAAAGAACAGGAGAAAGAGGAAATGCTTTTTC**T**ATGACGGTAGCTGACCAGAAAGCCACGGCTAACTACGT 480

No.15 401 TGTTGTCAGCAAAGAACAGGAGAAAGAGGAAATGCTTTTTC**T**ATGACGGTAGCTGACCAGAAAGCCACGGCTAACTACGT 480

No.19 401 TGTTGTCAGCAAAGAACAGGAGAAAGAGGAAATGCTTTTTC**T**ATGACGGTAGCTGACCAGAAAGCCACGGCTAACTACGT 480

No.30 401 TGTTGTCAGCAAAGAACAGGAGAAAGAGGAAATGCTTTTTC**T**ATGACGGTAGCTGACCAGAAAGCCACGGCTAACTACGT 480

No.31 401 TGTTGTCAGCAAAGAACAGGAGAAAGAGGAAATGCTTTTTC**T**ATGACGGTAGCTGACCAGAAAGCCACGGCTAACTACGT 480

IAM1676 512 GCCAGCAGCCGCGGTAATACGTAGGTGGCAAGCGTTGTCCGGATTTATTGGGCGTAAAGCGAGCGCAGGCGGTGATTTAA 591

No.1 481 GCCAGCAGCCGCGGTAATACGTAGGTGGCAAGCGTTGTCCGGATTTATTGGGCGTAAAGCGAGCGCAGGCGGTGATTTAA 560

No.3 481 GCCAGCAGCCGCGGTAATACGTAGGTGGCAAGCGTTGTCCGGATTTATTGGGCGTAAAGCGAGCGCAGGCGGTGATTTAA 560

No.13 481 GCCAGCAGCCGCGGTAATACGTAGGTGGCAAGCGTTGTCCGGATTTATTGGGCGTAAAGCGAGCGCAGGCGGTGATTTAA 560

No.15 481 GCCAGCAGCCGCGGTAATACGTAGGTGGCAAGCGTTGTCCGGATTTATTGGGCGTAAAGCGAGCGCAGGCGGTGATTTAA 560

No.19 481 GCCAGCAGCCGCGGTAATACGTAGGTGGCAAGCGTTGTCCGGATTTATTGGGCGTAAAGCGAGCGCAGGCGGTGATTTAA 560

No.30 481 GCCAGCAGCCGCGGTAATACGTAGGTGGCAAGCGTTGTCCGGATTTATTGGGCGTAAAGCGAGCGCAGGCGGTGATTTAA 560

No.31 481 GCCAGCAGCCGCGGTAATACGTAGGTGGCAAGCGTTGTCCGGATTTATTGGGCGTAAAGCGAGCGCAGGCGGTGATTTAA 560

IAM1676 592 GTCTGATGTGAAAGCCCCCAGCTCAACTGGGGAGGGTCATTGGAAACTGGATCACTTGAGTGCAGAA**G**AGGAGAGTGGAA 671

No.1 561 GTCTGATGTGAAAGCCCCCAGCTCAACTGGGGAGGGTCATTGGAAACTGGATCACTTGAGTGCAGAA**A**AGGAGAGTGGAA 640

No.3 561 GTCTGATGTGAAAGCCCCCAGCTCAACTGGGGAGGGTCATTGGAAACTGGATCACTTGAGTGCAGAA**A**AGGAGAGTGGAA 640

No.13 561 GTCTGATGTGAAAGCCCCCAGCTCAACTGGGGAGGGTCATTGGAAACTGGATCACTTGAGTGCAGAA**A**AGGAGAGTGGAA 640

No.15 561 GTCTGATGTGAAAGCCCCCAGCTCAACTGGGGAGGGTCATTGGAAACTGGATCACTTGAGTGCAGAA**A**AGGAGAGTGGAA 640

No.19 561 GTCTGATGTGAAAGCCCCCAGCTCAACTGGGGAGGGTCATTGGAAACTGGATCACTTGAGTGCAGAA**A**AGGAGAGTGGAA 640

No.30 561 GTCTGATGTGAAAGCCCCCAGCTCAACTGGGGAGGGTCATTGGAAACTGGATCACTTGAGTGCAGAA**A**AGGAGAGTGGAA 640

No.31 561 GTCTGATGTGAAAGCCCCCAGCTCAACTGGGGAGGGTCATTGGAAACTGGATCACTTGAGTGCAGAA**A**AGGAGAGTGGAA 640

IAM1676 672 TTCCATGTGTAGCGGTGAAATGCGTAGATATATGGAGGAACACCAGTGGCGAAGGCGGCTCTCTGGTCTGTAACTGACGC 751

No.1 641 TTCCATGTGTAGCGGTGAAATGCGTAGATATATGGAGGAACACCAGTGGCGAAGGCGGCTCTCTGGTCTGTAACTGACGC 720

No.3 641 TTCCATGTGTAGCGGTGAAATGCGTAGATATATGGAGGAACACCAGTGGCGAAGGCGGCTCTCTGGTCTGTAACTGACGC 720

No.13 641 TTCCATGTGTAGCGGTGAAATGCGTAGATATATGGAGGAACACCAGTGGCGAAGGCGGCTCTCTGGTCTGTAACTGACGC 720

No.15 641 TTCCATGTGTAGCGGTGAAATGCGTAGATATATGGAGGAACACCAGTGGCGAAGGCGGCTCTCTGGTCTGTAACTGACGC 720

No.19 641 TTCCATGTGTAGCGGTGAAATGCGTAGATATATGGAGGAACACCAGTGGCGAAGGCGGCTCTCTGGTCTGTAACTGACGC 720

No.30 641 TTCCATGTGTAGCGGTGAAATGCGTAGATATATGGAGGAACACCAGTGGCGAAGGCGGCTCTCTGGTCTGTAACTGACGC 720

No.31 641 TTCCATGTGTAGCGGTGAAATGCGTAGATATATGGAGGAACACCAGTGGCGAAGGCGGCTCTCTGGTCTGTAACTGACGC 720

IAM1676 752 TGAGGCTCGAAAGCGTGGGTAGCAAACAGGATTAGATACCCTGGTAGTCCACGCCGTAAACGATGAGTGCTAAGTGTTGG 831

No.1 721 TGAGGCTCGAAAGCGTGGGTAGCAAACAGGATTAGATACCCTGGTAGTCCACGCCGTAAACGATGAGTGCTAAGTGTTGG 800

No.3 721 TGAGGCTCGAAAGCGTGGGTAGCAAACAGGATTAGATACCCTGGTAGTCCACGCCGTAAACGATGAGTGCTAAGTGTTGG 800

No.13 721 TGAGGCTCGAAAGCGTGGGTAGCAAACAGGATTAGATACCCTGGTAGTCCACGCCGTAAACGATGAGTGCTAAGTGTTGG 800

No.15 721 TGAGGCTCGAAAGCGTGGGTAGCAAACAGGATTAGATACCCTGGTAGTCCACGCCGTAAACGATGAGTGCTAAGTGTTGG 800

No.19 721 TGAGGCTCGAAAGCGTGGGTAGCAAACAGGATTAGATACCCTGGTAGTCCACGCCGTAAACGATGAGTGCTAAGTGTTGG 800

No.30 721 TGAGGCTCGAAAGCGTGGGTAGCAAACAGGATTAGATACCCTGGTAGTCCACGCCGTAAACGATGAGTGCTAAGTGTTGG 800

No.31 721 TGAGGCTCGAAAGCGTGGGTAGCAAACAGGATTAGATACCCTGGTAGTCCACGCCGTAAACGATGAGTGCTAAGTGTTGG 800
